# Non-emphysematous chronic obstructive pulmonary disease is associated with diabetes mellitus

**DOI:** 10.1186/1471-2466-14-164

**Published:** 2014-10-24

**Authors:** Craig P Hersh, Barry J Make, David A Lynch, R Graham Barr, Russell P Bowler, Peter MA Calverley, Peter J Castaldi, Michael H Cho, Harvey O Coxson, Dawn L DeMeo, Marilyn G Foreman, MeiLan K Han, Benjamin J Harshfield, John E Hokanson, Sharon Lutz, Joe W Ramsdell, Elizabeth A Regan, Stephen I Rennard, Joyce D Schroeder, Frank C Sciurba, Robert M Steiner, Ruth Tal-Singer, Edwin JR van Beek, Edwin K Silverman, James D Crapo, the COPDGene and ECLIPSE Investigators

**Affiliations:** Channing Division of Network Medicine, Boston, MA USA; Division of Pulmonary and Critical Care Medicine, Brigham and Women’s Hospital, Boston, MA USA; Division of Pulmonary and Critical Care Medicine, National Jewish Health, Denver, CO USA; Department of Radiology, National Jewish Health, Denver, CO USA; Department of Medicine, Columbia University, New York, NY USA; Division of Infection and Immunity Clinical Sciences Centre, University Hospital Aintree, Liverpool, UK; Department of Radiology, University of British Columbia, Vancouver, Canada; Division of Pulmonary and Critical Care Medicine, Morehouse School of Medicine, Atlanta, GA USA; Division of Pulmonary and Critical Care Medicine, University of Michigan Health System, Ann Arbor, MI USA; Department of Epidemiology, Colorado School of Public Health, Aurora, CO USA; Department of Biostatistics, Colorado School of Public Health, Aurora, CO USA; Division of Pulmonary and Critical Care Medicine, University of California, San Diego, CA USA; Division of Pulmonary, Critical Care, Sleep and Allergy, University of Nebraska Medical Center, Omaha, NE USA; Division of Pulmonary, Allergy, and Critical Care Medicine, University of Pittsburgh, Pittsburgh, PA USA; Department of Radiology, Temple University, Philadelphia, PA USA; GlaxoSmithKline R&D, King of Prussia, PA USA; Department of Radiology, University of Edinburgh, Edinburgh, Scotland

**Keywords:** Airway disease, CT scan, Diabetes mellitus, Emphysema, Spirometry

## Abstract

**Background:**

Chronic obstructive pulmonary disease (COPD) has been classically divided into blue bloaters and pink puffers. The utility of these clinical subtypes is unclear. However, the broader distinction between airway-predominant and emphysema-predominant COPD may be clinically relevant. The objective was to define clinical features of emphysema-predominant and non-emphysematous COPD patients.

**Methods:**

Current and former smokers from the Genetic Epidemiology of COPD Study (COPDGene) had chest computed tomography (CT) scans with quantitative image analysis. Emphysema-predominant COPD was defined by low attenuation area at -950 Hounsfield Units (LAA_-950_) ≥10%. Non-emphysematous COPD was defined by airflow obstruction with minimal to no emphysema (LAA_-950_ < 5%).

**Results:**

Out of 4197 COPD subjects, 1687 were classified as emphysema-predominant and 1817 as non-emphysematous; 693 had LAA_-950_ between 5–10% and were not categorized. Subjects with emphysema-predominant COPD were older (65.6 vs 60.6 years, p < 0.0001) with more severe COPD based on airflow obstruction (FEV_1_ 44.5 vs 68.4%, p < 0.0001), greater exercise limitation (6-minute walk distance 1138 vs 1331 ft, p < 0.0001) and reduced quality of life (St. George’s Respiratory Questionnaire score 43 vs 31, p < 0.0001). Self-reported diabetes was more frequent in non-emphysematous COPD (OR 2.13, p < 0.001), which was also confirmed using a strict definition of diabetes based on medication use. The association between diabetes and non-emphysematous COPD was replicated in the ECLIPSE study.

**Conclusions:**

Non-emphysematous COPD, defined by airflow obstruction with a paucity of emphysema on chest CT scan, is associated with an increased risk of diabetes. COPD patients without emphysema may warrant closer monitoring for diabetes, hypertension, and hyperlipidemia and vice versa.

**Trial registration:**

Clinicaltrials.gov identifiers: COPDGene NCT00608764, ECLIPSE NCT00292552.

**Electronic supplementary material:**

The online version of this article (doi:10.1186/1471-2466-14-164) contains supplementary material, which is available to authorized users.

## Background

Chronic obstructive pulmonary disease (COPD) is a heterogeneous disease, including emphysema and large and small airway disease. The most recent update from the Global Initiative for Obstructive Lung Disease (GOLD) has addressed COPD subgroups [1], and there is a recognized need to better define COPD subtypes [2]. Several COPD subtypes have been shown to respond to specific treatments, including long-term oxygen for hypoxemic patients [3, 4], lung volume reduction surgery for upper lobe predominant emphysema [5], and medications including inhaled corticosteroids, azithromycin and roflumilast for frequent acute exacerbations [6–8].

Classic COPD subtypes include the “pink puffer” (underweight, emphysema, and normal resting oxygen saturation) and the “blue bloater” (overweight, chronic bronchitis, and hypoxemia) [9]. Though not appearing in the GOLD document and other management guidelines [1, 10], these subtypes persist in textbooks [11, 12]. The pink puffer – blue bloater distinction is vaguely defined and only applies to severe COPD patients, potentially limiting its clinical utility. However, the distinction between airway-predominant and emphysema-predominant COPD, which has some parallels to the pink puffer – blue bloater classification, may still have utility [13–17]. Historically, determinations about the extent of emphysema and airway disease could only be made on pathological specimens; however, chest computed tomography (CT) scans provide extensive anatomic information about COPD [18, 19]. As chest CT scans are becoming more frequently used for lung cancer screening and other indications [20, 21], these subtypes may be more easily identified in COPD patients.

The Genetic Epidemiology of COPD Study (COPDGene) has enrolled over 10,000 smokers with and without COPD across the United States [22]. The large sample size with extensive clinical data, including volumetric chest CT scans, allows for COPD subtyping. We aimed to create a simplified distinction between emphysema-predominant and presumed airway-predominant COPD based on the presence or absence of emphysema on chest CT scan. We hypothesized that these COPD subtypes would show associations with clinical characteristics and co-morbidities that have implications for the evaluation and management of patients with COPD. We assessed generalizability by replicating subtype associations in the Evaluation of COPD Longitudinally to Identify Predictive Surrogate End-points (ECLIPSE) Study [23].

## Methods

### Study subjects

COPDGene enrolled smokers with and without COPD at 21 clinical centers throughout the United States between 2007–2011 [22]. Subjects were self-classified non-Hispanic whites and non-Hispanic African Americans ages 45–80 with at least 10 pack-years of lifetime smoking. During the study visit, subjects underwent a limited physical examination, spirometry before and after inhaled bronchodilator, and a six-minute walk test to assess exercise capacity. Subjects completed questionnaires on respiratory disease, medical history and medications. The St. George’s Respiratory Questionnaire (SGRQ) measured disease-related quality of life [24]. Study protocols and questionnaires are available at http://www.copdgene.org. The ECLIPSE study is described in the Supplementary Methods (Additional file 1) [23]. COPDGene and ECLIPSE were approved by the institutional review boards at Partners Healthcare and all participating centers (Additional files 2 and 3). Subjects provided written informed consent.

### Chest CT scans

All subjects underwent a volumetric chest CT scan performed at full inspiration and at relaxed expiration. All chest CT scans were subjected to a standard quality control procedure. Quantitative image analysis was performed using 3D SLICER [25, 26] and VIDA Pulmonary Workstation software (Vida Diagnostics, Coralville, Iowa). Emphysema was quantified by the percent of lung voxels with attenuation ≤ -950 Hounsfield Units (HU) on inspiratory scan [27]. Subjects were considered to have emphysema-predominant COPD if this value exceeded 10%, corresponding to 3 standard deviations above the mean in normal non-smokers from COPDGene [28]. In contrast, non-emphysematous COPD was defined by minimal to absent emphysema, specifically <5% (mean +1 SD in normal non-smokers). Airway disease was assessed by the wall area percent of segmental airways and as the square root wall area of a hypothetical airway with 10 mm internal perimeter (SRWA-Pi10) [19].

### Statistical analysis

We used the COPDGene dataset version date 19-September-2012. The analysis was restricted to subjects with airflow obstruction, defined by FEV_1_/FVC <0.7 after bronchodilator, corresponding to GOLD stages 1–4 [1]. Based on expert opinion and previous COPDGene publications, we selected a set of clinical phenotypes and comorbidities to compare across the imaging subtypes (Table 1) [29–37]. Univariate comparisons used t-tests for continuous variables and chi-squared tests for binary variables. Logistic regression models were used to adjust for covariates including age, sex, race, body mass index, current smoking status, lifetime pack-years of smoking, and severity of airflow obstruction, assessed as post-bronchodilator FEV_1_ percent predicted. Stratified logistic regression models were adjusted for the same covariates excluding the quantitative of categorical variable that was used to define the strata (e.g. models in obese and non-obese subjects were not adjusted for BMI). Analyses were performed using R statistical software. Logistic regression analysis for diabetes in ECLIPSE is described in the Additional file 1.

**Table 1:** **COPD characteristics and comorbidity definitions**

Characteristic or comorbidity	Definition	COPDGene reference, if applicable
Asthma-COPD overlap	Self-report of physician diagnosis of asthma before age 40	Hardin 2011 [29]
Hypoxemia	Resting oxygen saturation ≤88%	Kim 2011 [30]
Frequent exacerbator	2 or more exacerbations requiring antibiotics and/or systemic steroids in the year prior to enrollment	Han 2011 [33]
Severe, early-onset COPD	Age < 55 years, FEV_1_ < 50% predicted	Foreman 2011 [34]
Poor exercise capacity	6-minute walk distance <500 feet	Rambod 2012 [32]
Bronchodilator response	Increase in FEV_1_ 200 ml and 12% from baseline	
Pink puffer	Emphysema > 10%, BMI ≤ 20, O_2_ sat ≥ 90%	
Blue bloater	Chronic bronchitis, BMI > 25, O_2_ sat < 90%	
Chronic bronchitis	Chronic cough and phlegm for ≥3 mo/yr for at least 2 consecutive years	Kim 2011 [31]
Chronic prednisone use	Self-report	Swift 2012 [35]
Cardiovascular disease	Self-report of heart attack, coronary artery disease, angina, angioplasty, coronary artery bypass graft, congestive heart failure, peripheral vascular disease, transient ischemic attack or stroke	Black-Shinn 2014 [36]
Coronary disease	Self-report of heart attack, coronary artery disease, angina, angioplasty, or coronary artery bypass graft	
Congestive heart failure	Self-report	
Peripheral vascular disease	Self-report	
Cerebrovascular disease	Self-report of transient ischemic attack or stroke	
Sleep apnea	Self-report	
Diabetes mellitus	Self-report	Kinney 2014 [37]
Metabolic syndrome	3 of 4: BMI > 30 (measured), diabetes mellitus, hypertension, and high cholesterol (all self-report)	
Gastroesophageal reflux disease	Self-report	
Peptic ulcer disease	Self-report	
Osteoporosis	Self-report	

## Results

**Figure 1:**
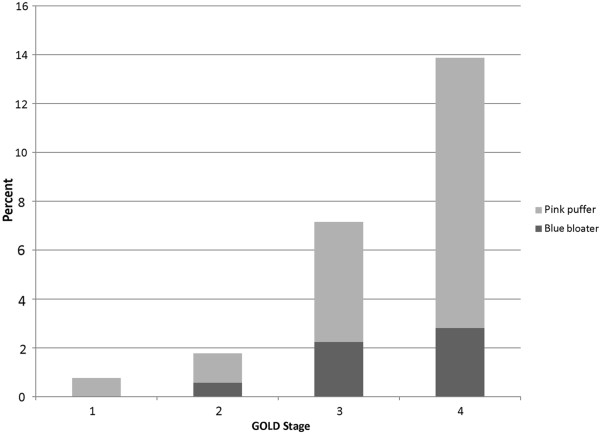
**Frequencies of the “pink puffer” and “blue bloater” subtypes of COPD by severity of airflow obstruction.** See Methods for definitions.

COPDGene enrolled 10,300 subjects. There were 4197 current and former smokers with airflow obstruction (FEV_1_/FVC < 0.7 on post-bronchodilator spirometry) and inspiratory chest CT scans passing quality control included in this analysis. Figure 1 demonstrates that the classic COPD subtypes of pink puffers and blue bloaters increased in frequency in more severe COPD, yet these subjects remained infrequent even among subjects with the most severe airflow obstruction (see Table 1 for definitions). Blue bloaters were less common than pink puffers across all level of lung function impairment.

**Figure 2:**
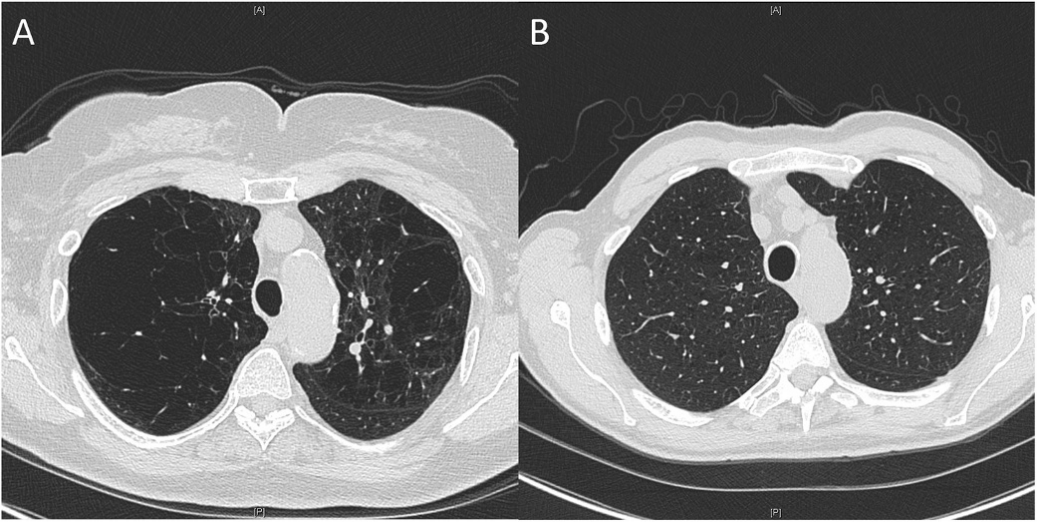
**Chest CT scans from COPDGene subjects demonstrating emphysema-predominant and non-emphysematous COPD. (A)** Emphysema-predominant: FEV_1_ 55.8% predicted, 29.0% emphysema. **(B)** Non-emphysematous: FEV_1_ 55.3% predicted, 4.2% emphysema.

Of the 4197 COPD subjects, 1687 subjects were defined as emphysema-predominant and 1817 as non-emphysematous (Table 2, Figure 2). 693 subjects had emphysema values between 5–10% and could not be classified into one of the two categories (Additional file 4: Table S1). Emphysema-predominant subjects had more severe COPD, based on lower lung function, reduced exercise capacity on 6-minute walk test, more severe dyspnea, and reduced quality of life (higher scores on SGRQ). Non-emphysematous subjects had greater bronchodilator responsiveness expressed as the absolute change in FEV_1_ and as the change in FEV_1_ as a percent of predicted FEV_1_, while emphysema-predominant subjects had greater change expressed as a percent of baseline FEV_1_. Non-emphysematous subjects had thicker airways. The characteristics of the unclassified subjects with 5–10% emphysema generally fell between the non-emphysematous and emphysema predominant subjects (Additional file 4: Table S1).

**Table 2:** **Demographic and clinical attributes of subjects with non-emphysematous and emphysema-predominant COPD (GOLD 1–4)**

	No/minimal emphysema	Emphysema-predominant	p-value
N	1817	1687	
Age	60.6 (±8.8)	65.6 (±7.7)	<0.0001
Male sex	933 (51.3%)	1000 (59.3%)	<0.0001
African American race	479 (26.4%)	288 (17.1%)	<0.0001
Pack-years of smoking	47.3 (±24.4)	55.9 (±28.1)	<0.0001
Current smoking	1122 (61.8%)	392 (23.2%)	<0.0001
Body Mass Index, kg/m^2^	29.3 (±6.3)	25.9 (±5.2)	<0.0001
Forced Expiratory Volume in 1 s (FEV_1_), % predicted	68.4 (±18.4)	44.5 (±21.0)	<0.0001
GOLD Stage			
1	487 (26.8%)	121 (7.2%)	
2	1025 (56.4%)	453 (26.9%)	
3	267 (14.7%)	649 (38.5%)	
4	38 (2.1%)	464 (27.5%)	
FEV_1_ / Forced Vital Capacity ratio	0.61 (±0.08)	0.42 (±0.12)	<0.0001
Bronchodilator response, change in FEV_1_, % of baseline	7.2 (±11.7)	9.1 (±12.6)	<0.0001
Bronchodilator response, change in FEV_1_, L	0.11 (±0.19)	0.09 (±0.13)	0.001
Bronchodilator response, % of predicted FEV_1_	3.8 (±6.4)	3.2 (±4.5)	0.002
6-minute walk distance, ft.	1331 (±383)	1138 (±403)	<0.0001
Oxygen saturation by pulse oximetry, %	95.9 (±2.8)	94.0 (±3.9)	<0.0001
Modified Medical Research Council dyspnea score	1.5 (±1.5)	2.4 (±1.3)	<0.0001
St. George’s Respiratory Questionnaire total score	30.7 (±22.9)	43.0 (±20.6)	<0.0001
BODE index*	1.6 (±1.7)	3.6 (±2.1)	<0.0001
Emphysema at -950 Hounsfield units, %	2.0 (±1.4)	23.6 (±10.7)	<0.0001
Square root wall area of an airway with 10 mm internal perimeter	3.71 (±0.15)	3.70 (±0.13)	0.001
Wall area % of segmental airways	62.7 (±3.4)	62.2 (±3.0)	<0.0001

*BODE = Body mass, airflow Obstruction, Dyspnea, Exercise capacity [38].

Mean (±SD) or N (%) are shown.

Table 3 shows the associations of the clinical characteristics and co-morbidities with the imaging subtypes. The COPD-asthma overlap group is more common in non-emphysematous COPD, as is bronchodilator responsiveness, when assessed using the American Thoracic Society/European Respiratory Society criteria [39]. Underweight subjects are more common among emphysema-predominant COPD. There was no difference in the prevalence of chronic bronchitis between the two imaging subtypes. Cardiovascular disease by various definitions, though statistically different between the imaging subtypes in the multivariate regression models, was not seen more frequently in non-emphysematous subjects, with the possible exception of congestive heart failure (Table 3). Both diabetes mellitus and the metabolic syndrome are significantly more common in non-emphysematous COPD (diabetes OR 2.13, p < 0.0001; metabolic syndrome OR 1.87, p < 0.0001). Using a stricter diabetes definition of self-report plus medication use (see methods in Additional file 1), there was a significant association with non-emphysematous COPD (OR 2.45, p < 0.0001). The associations with self-reported diabetes and the metabolic syndrome were similar in analyses restricted to subjects with FEV_1_ < 80% predicted (data not shown).

**Table 3:** **Associations of non-emphysematous and emphysema-predominant COPD with other clinical subgroups and comorbidities**

	Frequency		Logistic regression	
	Non-emphysematous	Emphysema-predominant	OR (adjusted)*	p-value
COPD/asthma overlap	281 (15.5%)	178 (10.6%)	1.64	0.0006
Hypoxemia	44 (2.4%)	159 (9.4%)	0.76	0.2
Frequent exacerbator	208 (11.4%)	330 (19.6%)	0.88	0.3
Severe, early-onset COPD	91 (5.0%)	111 (6.6%)	1.07	0.9
Poor exercise capacity	51 (2.8%)	119 (7.1%)	0.93	0.8
Bronchodilator response, ATS/ERS definition [39]	634 (34.9%)	569 (33.7%)	1.20	0.05
Chronic prednisone use	44 (2.4%)	124 (7.4%)	0.73	0.2
Low body mass index	59 (3.2%)	180 (10.7%)	0.39	<0.0001
Chronic bronchitis	470 (25.9%)	431 (25.5%)	0.87	0.2
Cardiovascular disease	393 (21.6%)	399 (23.7%)	1.44	0.0009
Coronary disease	277 (15.2%)	278 (16.5%)	1.36	0.01
Congestive heart failure	91 (5.0%)	59 (3.5%)	3.76	<0.0001
Peripheral vascular disease	58 (3.2%)	53 (3.1%)	1.59	0.07
Cerebrovascular disease	96 (5.3%)	102 (6.0%)	1.14	0.5
Sleep apnea	271 (14.9%)	199 (11.8%)	1.17	0.3
Diabetes mellitus	263 (14.5%)	146 (8.7%)	2.13	<0.0001
Metabolic syndrome	360 (19.8%)	173 (10.3%)	1.87	<0.0001
Gastroesophageal reflux disease	498 (27.4%)	511 (30.3%)	0.93	0.5
Stomach ulcers	147 (8.1%)	183 (10.8%)	0.76	0.07
Osteoporosis	278 (15.3%)	372 (22.1%)	0.96	0.7

*All models were adjusted for age, sex, race, pack-years, current smoking status, BMI, and FEV_1_% predicted, except low body mass index regression which was adjusted for the same covariates excluding BMI. Odds ratio is for non-emphysematous compared to emphysema-predominant COPD.

To ensure that the associations with diabetes and the metabolic syndrome were not due to confounding, we performed logistic regression analyses stratified by various potential confounding factors (Table 4 and Additional file 4: Table S2). The increased frequency of diabetes in non-emphysematous COPD persisted in stratified analyses of obese and non-obese subjects, current and former smokers, mild-moderate (GOLD 1–2) and severe-very severe COPD (GOLD 3–4), non-Hispanic white and African American subjects, and older and younger subjects (Table 4). Odds ratios were similar or increased in the stratified analyses using the stricter diabetes definition of self-report plus medication (data not shown). The associations with metabolic syndrome persisted in stratified analyses (Table S2).

**Table 4:** **Stratified logistic regression analyses for diabetes**

Variable	Stratum	Non-emphysematous	Emphysema-predominant	OR (adjusted)*	p-value
Obesity	BMI ≤ 30	89 (8.2%)	90 (6.6%)	1.82	0.004
	BMI > 30	174 (23.8%)	56 (17.0%)	2.71	<0.001
Current smoker	No	126 (18.1%)	123 (9.5%)	1.87	<0.001
	Yes	137 (12.2%)	23 (5.9%)	2.85	<0.001
GOLD Stage	1–2	191 (12.6%)	47 (8.2%)	1.87	0.001
	3–4	72 (23.6%)	99 (8.9%)	2.11	0.001
Race	Non-Hispanic white	185 (13.8%)	113 (8.1%)	2.23	<0.001
	African American	78 (16.3%)	33 (11.5%)	1.96	0.02
Age	<65	147 (11.8%)	53 (7.1%)	2.07	0.001
	≥65	116 (20.2%)	93 (9.9%)	2.16	<0.001

*Models were adjusted for age, sex, race, pack-years, current smoking status, BMI, and FEV_1_% predicted, excluding the variable that was used to define the strata (e.g. models in obese and non-obese subjects were not adjusted for BMI). Odds ratio is for non-emphysematous compared to emphysema-predominant COPD.

In ECLIPSE, there were 283 non-emphysema predominant and 1211 emphysema-predominant COPD subjects (Additional file 4: Table S3). Similar to COPDGene, emphysema-predominant subjects had more severe airflow obstruction. Diabetes was reported by 10.6% of non-emphysematous and 8.2% of emphysema-predominant subjects. A logistic regression model replicated the association between diabetes and non-emphysema predominant COPD (OR 1.62, 1-sided p-value = 0.034).

## Discussion

In the COPDGene Study, we defined subtypes of emphysema-predominant and non-emphysematous COPD based on a distinction between high and low emphysema on chest CT scan. We found an increased prevalence of diabetes in non-emphysematous COPD subjects, which was confirmed in the ECLIPSE study. Subjects with emphysema-predominant COPD had more severe airflow obstruction, but the proportions of subjects with frequent exacerbations or exercise intolerance were similar in the two subgroups when adjusted for differences in lung function. We confirmed expected associations of asthma-COPD overlap syndrome with non-emphysematous COPD and low BMI with emphysema-predominant COPD. The classic pink puffer – blue bloater subtypes were found in low frequencies in a modern COPD population. There was no difference in the rate of chronic bronchitis across the two subtypes, highlighting another shortcoming of this classic dichotomy. The pink puffer – blue bloater distinction is unlikely to be useful and should be abandoned.

The heterogeneous nature of COPD is increasingly recognized [2]. Previous efforts to identify subtypes of COPD have relied on statistical approaches such as cluster analysis and have identified clusters with emphysema and airway-predominant disease [40–42]. Garcia-Aymerich and colleagues found a cluster of subjects with milder COPD, obesity, diabetes, and cardiovascular disease, who had less emphysema in those subjects with CT scans [43]. These statistical approaches are promising, but they often require a large number of input variables and the output may be difficult to interpret. In our analysis, the simple distinction based on the presence or absence of emphysema yielded similar results and may be easier to apply clinically than the cluster-based methods.

Diabetes and the metabolic syndrome are frequent comorbidities in COPD patients [44–47]. COPD, diabetes, and the metabolic syndrome are all related to systemic inflammation, which may explain the co-occurrence. Studies examining whether inhaled corticosteroids increase risk of diabetes have shown conflicting results [48, 49]. Regardless, any association between ICS and diabetes would not explain the high frequency of the other components of the metabolic syndrome in COPD.

In a subset of COPDGene subjects, Han and colleagues examined CT features associated with COPD exacerbations [33]. They found that diabetes was more frequent in subjects with airway disease, defined by airway wall measures. The current study using all COPD subjects in COPDGene extends those results, using a paucity of emphysema on CT scan as a surrogate for airway-predominant COPD. The concordant results using a simple definition of airway-predominant COPD strengthens the current findings. Inflammation in emphysema may be different from inflammation in airway disease [50], explaining the variable correlations between these COPD phenotypes and other diseases associated with systemic inflammation. Analyses within well-characterized cohorts of COPD patients including biomarkers, chest CT scans and comorbidities would be required to clarify these inflammatory phenotypes.

The observation that non-emphysematous COPD is associated with diabetes may have clinical implications. Current guidelines from the U.S. Preventive Services Task Force (USPTF) and other organizations recommend routine screening for hypertension and hyperlipidemia in adults [51]. However, recommendations for diabetes screening vary. The USPTF recommends diabetes screening only in adults with blood pressure greater than 135/80 mmHg [52]. The American Diabetes Association recommends screening all adults who are overweight and have one or more risk factors for diabetes, as well as all adults beginning at age 45 [53]. One might consider adding COPD, specifically non-emphysematous COPD, to the list of diabetes risk factors. With the increasing use of chest CT scans for screening and diagnosis, physicians will increasingly be able to identify COPD patients with and without emphysema. Since patients with chronic diseases may be undertreated for other medical conditions [54], our results serve as a reminder to physicians to screen for diabetes, hypertension, and lipid disorders in patients with non-emphysematous COPD. Conversely, clinicians should obtain spirometry in current and former smokers with diabetes and/or metabolic syndrome who present with respiratory symptoms.

Besides a heightened awareness of disease screening in patients with non-emphysematous COPD, our findings may potentially affect treatment. For example, the phosphodiesterase-4 inhibitor roflumilast is indicated in severe COPD patients with chronic bronchitis and frequent acute exacerbations [8]. Wouters et al. have shown that roflumilast improves glucose control in subjects with newly-diagnosed diabetes without COPD [55]. The effect of roflumilast on both COPD and diabetes outcomes in subjects with both diseases warrants further study. Since we found that chronic bronchitis was equally frequent in both imaging subtypes, non-emphysematous COPD may define a more specific subgroup that might benefit from roflumilast than does chronic bronchitis. Other anti-inflammatory agents may also have beneficial effects in COPD subtypes. Our analysis has several limitations. We divided subjects into emphysema-predominant and non-emphysematous COPD, as a surrogate for airway-predominant COPD [17]. The non-emphysematous subgroup did have large airway disease on chest CT scans. However, the major site of airflow limitation is in the small airways. Measurements of small airways (<2 mm) are limited due to the spatial resolution of CT scans. Additionally, the emphysema-predominant subtype may still include substantial airway disease since we did not account for CT airway measurements when defining the subtypes. In order to be clinically-relevant, we created a simple distinction on chest CT scans which does not require complex airway measurements. The quantitative CT emphysema categories served as a surrogate for visual reading of the presence or absence of emphysema, as would be found in a clinical radiology report. Standardized radiologist readings of chest CT scans were not available in COPDGene. However, this simplified distinction proved to be generalizable to a second study population which included COPD subjects from multiple countries.

In COPDGene, comorbidities were defined by subject self-report. Previous studies have shown subject report of diabetes to be reliable [56–58]. In addition, we confirmed our findings using a strict definition of diabetes including medication usage. However, we could not apply strict definitions of metabolic syndrome, which would require measurements of waist circumference and triglyceride, HDL, and glucose levels [59]. Using more precise definitions may strengthen the associations found using self-reported data. Additionally, we used cross-sectional data, so we cannot determine the relative timing of the onset of non-emphysematous COPD and diabetes and the metabolic syndrome. Longitudinal data would be needed to address causality. COPDGene and ECLIPSE included non-Hispanic white and non-Hispanic African American subjects; it is not known whether our findings are generalizable to other racial and ethnic groups.

## Conclusions

Despite these limitations, we were able to create an imaging-based classification of emphysema-predominant and non-emphysematous COPD. The emphysema-predominant subjects had more severe COPD, based on measures of lung function, exercise capacity and symptoms. However, the non-emphysematous COPD subjects had an increased prevalence of diabetes and the metabolic syndrome, consistent with systemic inflammation. This finding may encourage clinicians to screen for diabetes and lipid abnormalities in COPD patients, specifically those without significant emphysema. With the increase in chest CT scans being performed for other indications, such as lung cancer screening, COPD patients will be more easily able to be assigned to these imaging subtypes. Conversely, patients with diabetes or metabolic syndrome who present with respiratory symptoms should undergo spirometry to evaluate for COPD. Future studies of existing and novel anti-inflammatory agents in COPD may find better results if targeted to COPD subjects without substantial emphysema.

## Electronic supplementary material

Additional file 1: **Supplementary Methods.** (DOCX 18 KB)

Additional file 2: **COPDGene Institutional Review Board approvals.** (DOC 41 KB)

Additional file 3: **ECLIPSE Institutional Review Board approvals.** (DOC 80 KB)

Additional file 4: Table S1: Demographic and clinical attributes of subjects with non-emphysematous and emphysema-predominant COPD (GOLD 1–4), along with unclassified COPD subjects (5–10% emphysema). **Table S2.** Stratified logistic regression analyses in COPDGene for metabolic syndrome. **Table S3.** ECLIPSE subjects with non-emphysematous and emphysema-predominant COPD (GOLD 2–4). (DOCX 19 KB)

## Abbreviations

BODE: Body mass index, airflow obstruction, dyspnea, and exercise capacity
COPD: Chronic obstructive pulmonary disease
COPDGene: Genetic epidemiology of COPD study
ECLIPSE: Evaluation of COPD longitudinally to identify predictive surrogate end-points
FEV_1_: Forced expiratory volume in 1 second
FVC: Forced vital capacity
GOLD: Global initiative for chronic obstructive lung disease
OR: Odds ratio
SGRQ: St. George’s respiratory questionnaire.
