# Supplementary material for: Non-emphysematous chronic obstructive pulmonary disease is associated with diabetes mellitus
Source: BMC Pulm Med. 2014 Oct 24;14:164. doi: 10.1186/1471-2466-14-164 (PMC4216374; doi:10.1186/1471-2466-14-164)
Supplement: Supplementary file 1 — Additional file 1: Supplementary Methods. (DOCX 18 KB) [file 12890_2014_599_MOESM1_ESM.docx]

Additional File 1

Supplementary Methods

Diabetes medication review

At the COPDGene visit, study staff manually entered subject medication lists. Free text medication profiles were analyzed by Levenshtein distance to systematically determine if each medication matched a list of diabetes medications. All participants whose profile was labeled as unsure for diabetes (n=5) or whose self report did not agree with their drug profile (n=518; 457 reported diabetes but no medications, 61 reported diabetes medications but no self report of disease) and a 5% sample whose self report did agree (n=488) were reviewed by a physician, and no reversals of medication-inferred diabetes status were noted. Statistical analyses were repeated using a strict definition of diabetes that included both self report and medication use.

Validation study

The ECLIPSE (Evaluation of COPD Longitudinally to Identify Predictive Surrogate End-points) Study was a 3-year observational study of 2083 smokers with COPD (GOLD stages 2-4), 332 control smokers and 237 non-smoking controls [[1](#_ENREF_1)]. In ECLIPSE, diabetes and other co-morbidities were assessed by questionnaires. Similar to COPDGene and a previous ECLIPSE publication [[2](#_ENREF_2)], emphysema-predominant COPD was defined by >10% of lung voxels with attenuation ≤ -950 HU on inspiratory chest CT scans. Non-emphysematous COPD was defined as in COPDGene. Metabolic syndrome could not be assessed based on ECLIPSE questionnaire data. Study subjects provided written informed consent. ECLIPSE was approved by the institutional review boards at Partners Healthcare and all participating centers.

References

1. Vestbo J, Anderson W, Coxson HO, Crim C, Dawber F, Edwards L, Hagan G, Knobil K, Lomas DA, MacNee W *et al*: **Evaluation of COPD Longitudinally to Identify Predictive Surrogate End-points (ECLIPSE)**. *Eur Respir J* 2008, **31**(4):869-873.

2. Vestbo J, Edwards LD, Scanlon PD, Yates JC, Agusti A, Bakke P, Calverley PM, Celli B, Coxson HO, Crim C *et al*: **Changes in forced expiratory volume in 1 second over time in COPD**. *N Engl J Med* 2011, **365**(13):1184-1192.
