# Supplementary material for: Non-emphysematous chronic obstructive pulmonary disease is associated with diabetes mellitus
Source: BMC Pulm Med. 2014 Oct 24;14:164. doi: 10.1186/1471-2466-14-164 (PMC4216374; doi:10.1186/1471-2466-14-164)
Supplement: Supplementary file 3 — Additional file 3: ECLIPSE Institutional Review Board approvals. (DOC 80 KB) [file 12890_2014_599_MOESM3_ESM.doc]

| **Inv/Site No.** | **Institution & Address** | **IEC/IRB Committee** |
| --- | --- | --- |
| 027904/023622 | Asthma Centre  Ivan Vazov Street Nr 31  PO Box 1018  Pleven, 5800 Bulgaria | Ethics Committee for Multicentre Trials  8, Damyan Gruev str.,  Sofia 1303  Bulgaria |
| 076209/023623 | Military Medical Academy  Georgi Sofiiski 3 str  Sofia 1606 Bulgaria | Ethics Committee for Multicentre Trials  8, Damyan Gruev str.,  Sofia 1303  Bulgaria |
| 006269/023794 | Montreal Chest Institute  3650 St-Urbain, Room K307  Montreal, QC H2X 2P4 Canada | McGill University Health Center  Research Ethics Board  3650 St Urbain  Montreal, QC H2X 2P4 |
| 006610/023668 | The Lung Center  2775 Laurel St., 7^th^ Floor  Vancouver, BC V5Z 1M9 Canada | The University of British Columbia  Office of Research Services Clinical Research Ethics Board  Room210, 828 West 10^th^ Ave  Vancouver, BC V5Z 1L8 Canada |
| 004970/023483 | Queen Elizabeth II Health Sciences Centre  Halifax Infirmary  1796 Summer St., Room 5452  Halifax, NS B3H 3A7 Canada | **Capital Health Research Ethics Board** Centre for Clinical Research Building  118-5790 University Avenue  Halifax, NS B3H 1V7 Canada |
| 029347/023796 | McMaster University, Health Sciences Center  1200 Main St. West, Room 3U25  Hamilton, ON L8N 3Z5 Canada | Hamilton Health Sciences/Faculty of Health Sciences Research Ethics Board  293 Wellington St N, Suite 102 Hamilton, Ontario L8L 8E7 Canada |
| 035031/023795 | Pacific Lung Health Center  1081 Burrard Street  8B Providence Wing  Vancouver, BC V6Z 1Y6 Canada | **Office of Research Services (ORS)**  Providence Health Care Research Institute  Room 1125, 11th Floor  1190 Hornby Street c/o 1081 Burrard Street Vancouver, BC V6Z 1Y6 |
| 006193/023547 | Hopital Laval, Recherche Clinic  Centre de Pneumologie  2725 Chemin Sainte Foy  Pavillion U, Locale U 1751  Sainte Foy, QC G1V 4G5 Canada | Comité d’éthique de la recherche  Institut Universitaire de Cardiologie et de Pneumologie de Québec (IUCPQ)  2725, chemin Ste-Foy  Quebec, Qc  Canada  G1V 4G5 |
| 004395/02409 4 | Kingston General Hospital  Richardson House  102 Stuart Street  Kingtson, ON K7L 2V6 Canada | Queens University  Office of Research Services  Fleming Hall, Jemmett Wing, Room 301  Queens University  Kingston, ON, Canada |
| 000309/024204 | SPLiN s.r.o.  Oddeleni TRN  Cimicka 37/446  Praha 8 18200 Czech Republic | Multicentric Ethics Committee Fakultni nemocnice v Motole  V Uvalu 84 Prague 5 ZIP: 150 06  Czech Republic |
| 000683/023960 | H:S Hvidovre Hospital  Hjerte-Lungemedicinsk afdeling  Kettegaard Alle 30  Opgang 1  Hvidovre 2650 Denmark | Den videnskabsetiske komité for region hovedstaden  Regionsgaarden  Kongensvænge 2  3400 Hillerød |
| 001687/024403 | Astmacentrum Hornerheide  Hornerheide 1  Horn 6085 NM Netherlands | METC Zuidwest-Holland  M.H.H.A. Kirkels-Breukers  P.O Box 5011  2600 GA Delft  The Netherlands |

| 082272/023579 | Haukeland Universitets sykehus  Chest department  Jonas Liesvei 65  Bergen N 5021 Norway | | Regional Ethic Committee West  Haukeland University Hospital, N-5021 Bergen, Norway | | |
| --- | --- | --- | --- | --- | --- |
| 014566/024144 | P3 Research Bown Hospital  Churchill Drive Crofton Downs  Wellington 6035 New Zealand | | c/- Ministry of Health 1-3 The Terrace Level 1 Wellington 6011 | | |
| 136098/024146 | KOPA Golnik  Golnik 36  4204 Golnik Slovenia | | The National Medical Ethics Committee of the Republic of Slovenia  University Institute of Clinical Neurophysiology, Medical Center Ljubljana,  Zaloška c. 7, SI-1525 Ljubljana | | |
| 108244/026658 | Hospital Son Dureta  C/ Andrea Doria 55  Palma de Mallorca 07014 Spain | | Comité ètic d´investigació clínica Illes Balears  Conselleria de Salut i Consum Direcció General d'Avaluació i Acreditació Comitè Ètic d'Investigació Clínica de les Illes Balears (CEIC-IB) Camí de Jesús, 38 A 07011 Palma - Illes Balears | | |
| 000473/023973 | Aintree University Hospitals NHS Foundation Trust  Respiratory Research Department  Longmoor Lane, Ward 14a  Liverpool L9 7AL United Kingdom | | Oxfordshire REC C  2^nd^ Floor, Astral House  Chaucer Business Park  Granville Way  Bicester OX26 4JT | | |
| 082424/023706 | Cambridge Institute for Medical Research  Department of Medicine  Hills Road, Wellcome Trust / MRC Building  Cambridge CB2 2XY United Kingdom | | Oxfordshire REC C  2^nd^ Floor, Astral House  Chaucer Business Park  Granville Way  Bicester OX26 4JT | | |
| 029855/023707 | New Royal Infirmary of Edinburgh  Little France Crescent, Old Dalkeith Road  51 Little France Crescent  Edinburgh Midlothian EH16 4SA  United Kingdom | | Oxfordshire REC C  2^nd^ Floor, Astral House  Chaucer Business Park  Granville Way  Bicester OX26 4JT | | |
| 023731/023974 | Wythenshawe Hospital  Medicine Evaluation Unit  Southmoor Road  The Langley Building, North West Lung Research Centre  Manchester M23 9LT United Kingdom | | Oxfordshire REC C  2^nd^ Floor, Astral House  Chaucer Business Park  Granville Way  Bicester OX26 4JT | | |
| 029742/024037 | The Royal Free Hospital  Academic Unit of Respiratory Medicine  Pond Street  London NW3 2QG United Kingdom | | Oxfordshire REC C  2^nd^ Floor, Astral House  Chaucer Business Park  Granville Way  Bicester OX26 4JT | | |
| 001069/024393 | Institute of Phthisiatry and Pulmonology  Department of Pulmonology  10, Amosova Str  Kiev 03680 Ukraine | |  | | |
| 001047/024392 | Institute of Phthisiatry and Pulmonology  Department of Pulmonology  10, Amosova Str  Kiev 03680 Ukraine | |  | | |
| 001071/024362 | Institute of Phthisiatry and Pulmonology  Department of Pulmonology  10, Amosova Str  Kiev 03680 Ukraine | |  | | |
| 001063/024364 | Donetsk State Medical University  Department of Therapy  16 Illicha prospect  Donetsk 83003 Ukraine | |  | |  |
| 044783/023140 | University of Texas Health Science Center  Pulmonary Diseases  7400 Merton Minter Blvd., (111E)  San Antonio, TX 78229 United States | | University of Texas Health Science Center  7703 Floyd Curl Drive, Mail Code 7830  San Antonio, TX 78229-3900 | |  |
| 077534/023146 | Rhode Island Hospital  Division of Pulmonary, Sleep & Critical Care Medicine  593 Eddy Street, APC 7^th^ Floor  Providence, RI 02903 United States | | Lifespan Office of Research Administration  167 Point Street  Providence, RI 02903 | |  |
| 013075/023147 | Los Angeles Biomedical Research Institute at Harbor-UCLA Medical Center  Rehab Clinical Trials Center  1124 W. Carson St., Bldg. J4  Torrance, CA 90502 United States | | John F. Wolf, MD Human Subjects Committee  Los Angeles Biomedical Research Institute at Harbor-UCLA Medical Center  1124 West Carson Street  Torrance, CA 90502 | |  |
| 008578/023354. | St. Elizabeth’s Medical Center  Pulmonary STN-3  736 Cambridge Street  Boston, MA 02135 Unites States | | Research/Human Subjects Committee  Caritas St. Elizabeth's Medical Center Cambridge St., HOQ3 Boston, MA 02135 | |  |
| 021992/023148 | Pulmonary Associates of Richmond, Inc.  1000 Boulders Parkway, Suite 201  Richmond, VA 23225 United States | | Goodwyn Institution Review Board  9380 Main Street  Cincinnati, OH 45242 | |  |
| 010875/023149 | Pulmonary Associates, PA  1112 East McDowell Road  Phoenix, AZ 85006 United States | | Goodwyn Institution Review Board  9380 Main Street  Cincinnati, OH 45242 | |  |
| 011553/023150 | Advances in Medicine  42362 Bob Hope Drive  Rancho Mirage, CA 92270 Unites States | | Western International Review Board  3535 Seventh Ave SW  Olympia, WA 98508 | |  |
| 010094/023355 | Baylor Clinic-Baylor College of Medicine  6620 Main Street  Suite 11B, 16  Houston, TX 77030 United States | Baylor College of Medicine IRB  Clinical Research Studies  One Baylor Plaza, Mail stop 600D  Houston, TX 77030 | |  | |
| 015497/023356 | Dartmouth-Hitchcock Medical Center  Pulmonary & Critical Care Center  One Medical Center Drive  Lebanon, NH 03756 Unites States | Dartmouth-Hitchcock Medical Center  Committee for the Protection of Human Subjects  11 Rope Ferry Road #6210  Hanover, NH 03755 | |  | |
| 008005/023357 | National Jewish Medical & Research Center  Weinberg Clinical Research Unit  1400 Jackson Street  Denver, CO 80206 United States | National Jewish Medical & Research Center IRB  1400 Jackson Street  Denver, CO 80206 | |  | |
| 009021/023358 | University of Nebraska Medical Center  Pulmonary Clinical Studies Unit  982465 Nebraska Medical Center  DRC II1022  Omaha, NE 68198 United States | University of Nebraska Medical Center IRB  Academic & Research Services Bldg. 3000  987830 Nebraska Medical Center  Omaha, NE 68198 | |  | |
| 083482/023571 | Yale University School of Medicine  Internal Medicine/Pulmonary  1 Gilbert Street, TAC S 441  New Haven, CT 06520 United States | Yale University School of Medicine  Human Investigation Committee  47 College Street, Suite 204  New Haven, CT 06520 | |  | |
| 015449/023359 | Mayo Clinic  Pulmonary Clinical Research Center  Lanmark 2-46  14 – 2^nd^ Street SW  Rochester, MN 55905 United States | Mayo Foundation IRB  201 Building, Room 4-60 200 First Street SW Rochester, MN 55905 | |  | |
| 080801/023389 | Creighton University Medical Center  Pulmonary & Critical Care Division  601 N. 30^th^ Street, Suite 3820  Omaha, NE 68131 United States | | Creighton University Medical Center IRB  2500 California Plaza  Omaha, NE 68178 |  | |
| 010532/023489 | University of Pittsburgh Medical Center  Emphysema Research Center  3471 5^th^ Ave., Suite 1211  Pittsburgh, PA 15213 United States | | University of Pittsburgh IRB  3500 Fifth Ave, Ground Level  Pittsburgh, PA 15213 | |  |

| 021093/023390 | Houston VA Medical Center  2002 Holcombe Blvd.  Pulmonary 111-1, Room 3C-220  Houston, TX 77030 United States | Baylor College of Medicine IRB  Clinical Research Studies  One Baylor Plaza, Mail stop 600D  Houston, TX 77030 |
| --- | --- | --- |
| 008864/023392 | Midwest Chest Consultants, PC  330 First Capital Drive, Suite 470  St. Charles, MO 63301 United States | Goodwyn Institution Review Board  9380 Main Street  Cincinnati, OH 45242 |
| 077533/023391 | Harvard University–Brigham & Women’s Hospital  Channing Laboratory  181 Longwood Ave.  Boston, MA 02115 United States | Brigham & Women’s Hospital IRB  Partners Human Research Office  116 Huntington Ave, Suite 1002  Boston, MA 02116 |
| 016586/023393 | University of Miami School of Medicine  1600 NW 10th Ave, #7064-A (R-47)  Miami, FL 33136 | Western International Review Board  3535 Seventh Ave SW  Olympia, WA 98508 |
| 057745/023394 | Johns Hopkins Asthma & Allergy Center  5501 Hopkins Bayview Circle, Room 3B-58  Baltimore, MD 21224 United States | John Hopkins School of Medicine  Office of Human Subject Research  1620 McElderry St., Reed Hall  Suite B 130  Baltimore, MD 21205 |
| 012252/023395 | St. Francis Hospital & Medical Center  Pulmonary Medicine  114 Woodland Street  Hartford, CT 06105 United States | St. Francis Hospital & Medical Center IRB  Department of Research  114 Woodland St.  Hartford, CT 06105 |
