# Supplementary material for: Non-emphysematous chronic obstructive pulmonary disease is associated with diabetes mellitus
Source: BMC Pulm Med. 2014 Oct 24;14:164. doi: 10.1186/1471-2466-14-164 (PMC4216374; doi:10.1186/1471-2466-14-164)
Supplement: Supplementary file 4 — Additional file 4: Table S1: Demographic and clinical attributes of subjects with non-emphysematous and emphysema-predominant COPD (GOLD 1–4), along with unclassified COPD subjects (5–10% emphysema). Table S2. Stratified logistic regression analyses in COPDGene for metabolic syndrome. Table S3. ECLIPSE subjects with non-emphysematous and emphysema-predominant COPD (GOLD 2–4). (DOCX 19 KB) [file 12890_2014_599_MOESM4_ESM.docx]

Additional File 4

Table S1: Demographic and clinical attributes of subjects with non-emphysematous and emphysema-predominant COPD (GOLD 1-4), along with unclassified COPD subjects (5-10% emphysema). Mean (± SD) or N (%) are shown.

|  | No/minimal emphysema | Unclassified (5-10% emphysema) | Emphysema-predominant |
| --- | --- | --- | --- |
| N | 1817 | 693 | 1687 |
| Age | 60.6 (±8.8) | 63.9 (±8.5) | 65.6 (±7.7) |
| Male sex | 933 (51.3%) | 428 (61.8%) | 1000 (59.3%) |
| African American race | 479 (26.4%) | 147 (21.2%) | 288 (17.1%) |
| Pack-years of smoking | 47.3 (±24.4) | 52.5 (±29.4) | 55.9 (±28.1) |
| Current smoking | 1122 (61.8%) | 306 (44.2%) | 392 (23.2%) |
| Body Mass Index, kg/m^2^ | 29.3 (± 6.3) | 28.6 (±6.0) | 25.9 (± 5.2) |
| Forced Expiratory Volume in 1 s (FEV_1_), % predicted | 68.4 (± 18.4) | 62.7 (±20.8) | 44.5 (± 21.0) |
| GOLD Stage  1  2  3  4 | 487 (26.8%)  1025 (56.4%)  267 (14.7%)  38 (2.1%) | 152 (21.9%)  338 (48.8%)  162 (23.4%)  41 (5.9%) | 121 (7.2%)  453 (26.9%)  649 (38.5%)  464 (27.5%) |
| FEV_1_ / Forced Vital Capacity ratio | 0.61 (± 0.08) | 0.55 (±0.11) | 0.42 (± 0.12) |
| Bronchodilator response, change in FEV_1_, % of baseline | 7.2 (± 11.7) | 8.7 (±12.3) | 9.1 (± 12.6) |
| Bronchodilator response, change in FEV_1_, L | 0.11 (± 0.19) | 0.11 (±0.17) | 0.09 (± 0.13) |
| Bronchodilator response, % of predicted FEV_1_ | 3.8 (± 6.4) | 4.0 (±5.6) | 3.2 (± 4.5) |
| 6-minute walk distance, ft. | 1331 (± 383) | 1282 (±403) | 1138 (± 403) |
| Oxygen saturation by pulse oximetry, % | 95.9 (± 2.8) | 95.4 (±3.1) | 94.0 (± 3.9) |
| Modified Medical Research Council dyspnea score | 1.5 (± 1.5) | 1.8 (±1.5) | 2.4 (± 1.3) |
| St. George’s Respiratory Questionnaire total score | 30.7 (± 22.9) | 34.9 (±23.6) | 43.0 (± 20.6) |
| BODE index* | 1.6 (± 1.7) | 2.1 (±1.9) | 3.6 (± 2.1) |
| Emphysema at -950 Hounsfield units, % | 2.0 (± 1.4) | 7.3 (±1.5) | 23.6 (± 10.7) |
| Square root wall area of an airway with 10mm internal perimeter | 3.71 (± 0.15) | 3.69 (±0.15) | 3.70 (± 0.13) |
| Wall area % of segmental airways | 62.7 (± 3.4) | 62.2 (±3.2) | 62.2 (± 3.0) |

*BODE = Body mass, airflow Obstruction, Dyspnea, Exercise capacity [[60](#_ENREF_60)]

Table S2: Stratified logistic regression analyses in COPDGene for metabolic syndrome

| **Variable** | **Stratum** | **Non-emphysematous** | **Emphysema-predominant** | **OR (adjusted)*** | **p-value** |
| --- | --- | --- | --- | --- | --- |
| Obesity | BMI≤30 | 47 (4.3%) | 44 (3.2%) | 2.37 | 0.003 |
|  | BMI>30 | 313 (42.9%) | 129 (39.2%) | 1.86 | <0.001 |
| Current smoker | No | 200 (28.8%) | 149 (11.5%) | 1.74 | 0.001 |
|  | Yes | 160 (14.3%) | 24 (6.1%) | 2.11 | 0.007 |
| GOLD Stage | 1-2 | 264 (17.5%) | 67 (11.7%) | 1.60 | 0.008 |
|  | 3-4 | 96 (31.5%) | 106 (9.5%) | 2.01 | 0.002 |
| Race | Non-Hispanic white | 263 (19.7%) | 148 (10.6%) | 1.52 | 0.008 |
|  | African American | 97 (20.3%) | 25 (8.7%) | 4.18 | <0.001 |
| Age | <65 | 194 (15.6%) | 56 (7.5%) | 1.80 | 0.008 |
|  | ≥65 | 166 (29.0%) | 117 (12.5%) | 1.81 | 0.001 |

*Models were adjusted for age, sex, race, pack-years, current smoking status, BMI, and FEV_1_% predicted, excluding the variable that was used to define the strata (e.g. models in obese and non-obese subjects were not adjusted for BMI). Odds ratio is for non-emphysematous compared to emphysema-predominant COPD.

Table S3: ECLIPSE subjects with non-emphysematous and emphysema-predominant COPD (GOLD 2-4). Mean (± SD) or N (%) are shown.

|  | No/minimal emphysema | Emphysema-predominant | p-value |
| --- | --- | --- | --- |
| N | 283 | 1211 |  |
| Age | 61.0 (±7.9) | 63.9 (±6.6) | <0.0001 |
| Male sex | 142 (50.2%) | 814 (67.2%) | <0.0001 |
| Pack-years of smoking | 43.8 (±25.9) | 49.6 (±26.6) | 0.001 |
| Current smoking | 172 (60.8%) | 350 (28.9%) | <0.0001 |
| Body Mass Index, kg/m^2^ | 29.3 (± 6.3) | 25.9 (± 5.2) | <0.0001 |
| FEV_1_ % predicted | 59.4 (± 13.1) | 44.0 (± 14.7) | <0.0001 |
| GOLD Stage  2  3  4 | 214 (75.6%)  63 (22.3%)  6 (2.1%) | 391 (32.3%)  602 (49.7%)  218 (18.0%) |  |
